# Impact of CYP Enzyme Polymorphisms on Opioid Response in Anesthesia and Pain Medicine

**DOI:** 10.1155/prm/6605418

**Published:** 2026-09-06

**Authors:** Rongrong Yan, Yingyao Quan, Junyang Ma, Jing Cheng, Mengjiao Wan

**Affiliations:** ^1^ Department of Anesthesiology, Maternal and Child Health Hospital of Hubei Province, No.745 Wuluo Street Hongshan District, Wuhan 430070, Hubei, China

**Keywords:** CYP enzyme polymorphism, drug metabolism, opioids, pain medicine, personalized analgesia, pharmacogenomics

## Abstract

Opioids serve as the cornerstone for anesthesia, postoperative analgesia, and chronic pain management. However, significant interindividual variations exist in their efficacy and safety, posing substantial challenges to clinical practice. Genetic polymorphisms of Cytochrome P450 (CYP) enzymes, particularly CYP2D6 and CYP3A4/5, represent one of the primary contributors to these differences. This review aims to elucidate the mechanisms by which CYP enzyme gene polymorphisms influence opioid metabolism and discuss their clinical implications. Key conclusions indicate that the CYP2D6 genotype significantly influences the bioactivation of precursor drugs such as codeine and tramadol; ultrarapid metabolizers (UMs) are at substantial risk of toxicity when using codeine, whereas slow metabolizers (PM) are at increased risk of inadequate analgesia. Polymorphisms in CYP3A4/5 primarily affect the metabolism of agents such as fentanyl and sufentanil via drug–drug interactions, although evidence supporting genotype‐guided dose adjustments independent of clinical factors remains insufficient. Additionally, CYP2B6 polymorphisms are critical for methadone metabolism. Although integrating pharmacogenomic testing into clinical practice holds significant potential, current evidence supports selective testing in specific clinical scenarios rather than routine implementation across all opioid prescriptions.

## 1. Introduction

Opioids have long been central to anesthesia and pain management due to their potent analgesic effects. However, significant interindividual variability in opioid response remains a major clinical challenge, often attributed to differences in drug metabolism. Cytochrome P450 (CYP) enzymes, particularly CYP2D6 and CYP3A4/5, critically mediate the metabolism of many opioids [1, 2]. Genetic polymorphisms in these enzymes can result in variations in drug efficacy and toxicity, thereby influencing therapeutic outcomes [3]. The clinical significance of CYP2D6 in opioid metabolism has been extensively characterized, with recent work highlighting its role in personalized analgesia [4] and informing clinical practice guidelines [5, 6]. This narrative review aims to synthesize and critically evaluate the current literature on how CYP enzyme polymorphisms affect opioid metabolism, discuss their clinical implications, and outline future research directions. Furthermore, this review emphasizes the distinction between robust clinical recommendations and preliminary findings.

## 2. Methods

This article is a narrative review. To prepare this work, we conducted literature searches in the PubMed and Web of Science databases covering the period from 2020 to 2024. The search keywords included “CYP2D6 polymorphism,” “CYP3A4/5 polymorphism,” “CYP2B6 polymorphism,” “opioid metabolism,” “pharmacogenomics,” “personalized medicine,” “pain management,” and “anesthesia.” The inclusion criteria were peer‐reviewed original studies, reviews, and guidelines focusing on the relationship between CYP gene polymorphisms and opioid response. We prioritized clinical guidelines from authoritative organizations such as the Clinical Pharmacogenomics Implementation Consortium (CPIC) and the Dutch Pharmacogenomics Working Group (DPWG).

## 3. CYP Enzymes in Opioid Metabolism

### 3.1. CYP2D6: The Primary Metabolizer of Prodrugs

CYP2D6 is a critical enzyme in the metabolism of several opioids, playing a pivotal role in determining their pharmacokinetics and pharmacodynamics. It is particularly important for the bioactivation of prodrugs such as codeine and tramadol, catalyzing their conversion into active metabolites morphine and O‐desmethyltramadol, respectively, which are largely responsible for their analgesic effects [4, 5]. Conversely, while CYP2D6 metabolizes morphine, its primary metabolism is mediated by glucuronidation via UGT enzymes. For opioids such as oxycodone and hydrocodone, CYP2D6 contributes to the formation of active metabolites, but it is not the sole determinant of the analgesic response [6, 7].

The activity of CYP2D6 is highly variable among individuals due to genetic polymorphisms, leading to distinct phenotypes such as poor metabolizers (PMs), intermediate metabolizers (IMs), extensive metabolizers (EMs), and ultrarapid metabolizers (UMs) [8]. PMs exhibit reduced or absent CYP2D6 activity, leading to impaired conversion of prodrugs and potentially inadequate analgesia [9]. UMs, with multiple functional copies of the CYP2D6 gene, metabolize prodrugs much faster. The strongest clinical evidence for CYP2D6 UM‐related severe toxicity concerns codeine, where rapid conversion to morphine can lead to excessive plasma concentrations and increased risk of adverse effects such as respiratory depression [10–12]. Extrapolation of these UM‐related toxicity concerns to other opioids, such as oxycodone or hydrocodone, should be approached with caution, as CYP2D6’s role often involves generating active metabolites rather than being the sole pathway for the parent drug’s efficacy. Recent studies underscore the clinical relevance of CYP2D6 genotyping. For example, Saab et al. demonstrated that genetic testing for CYP2D6 polymorphisms can help predict individual responses to codeine [13]. Similarly, Deodhar et al. emphasized the role of CYP2D6 in hydrocodone and oxycodone metabolism, suggesting that genotype‐guided dosing could improve pain management outcomes [7]. The influence of CYP2D6 extends to addiction treatment, where its activity affects methadone metabolism [14]. Research indicates that individuals with certain CYP2D6 genotypes may require adjusted methadone doses to achieve optimal therapeutic effects [15].

### 3.2. CYP3A4/5: Key Regulators of Bioavailability and Drug Clearance

CYP3A4 and CYP3A5 are pivotal enzymes responsible for metabolizing several opioids, particularly fentanyl, sufentanil, methadone, and buprenorphine [16]. They play a critical role in regulating the bioavailability of these drugs by converting them into metabolites with reduced or negligible activity, thereby shaping their pharmacokinetic profile, efficacy, and potential toxicity. Recent evidence highlights the importance of CYP3A4/5 polymorphisms in dictating interindividual variability in drug response, underscoring the need for precision medicine strategies in opioid therapy [17].

#### 3.2.1. Role of CYP3A4/5 in Opioid Metabolism

Primarily expressed in the liver and small intestine, CYP3A4/5 contributes significantly to the first‐pass metabolism of orally administered opioids [16]. In the case of fentanyl, CYP3A4/5 catalyzes the formation of norfentanyl and other metabolites, reducing systemic drug availability [18]. Similarly, sufentanil is metabolized by CYP3A4/5 into less potent compounds [19, 20].

The activity of these enzymes varies considerably among individuals due to genetic polymorphisms, leading to differences in enzyme expression and catalytic efficiency. This variability results in altered drug clearance and systemic exposure. Individuals with reduced CYP3A4/5 activity (e.g., CYP3A5^∗^3/3 genotype) may experience increased opioid levels, potentially enhancing analgesia but also elevating the risk of adverse effects such as respiratory depression [21–23]. Conversely, those with high enzyme activity (e.g., CYP3A4 1/1 and CYP3A5 1/^∗^1 genotypes) may metabolize opioids more rapidly, potentially resulting in subtherapeutic drug levels and inadequate pain relief [24–26].

#### 3.2.2. Clinical Implications of CYP3A4/5 Polymorphisms

The clinical relevance of CYP3A4/5 polymorphisms is increasingly recognized. A study by Smith et al. demonstrated that genetic variations in CYP3A4 significantly influence fentanyl pharmacokinetics, with certain genotypes associated with reduced clearance and increased risk of respiratory depression [27]. Similarly, Johnson et al. reported that individuals with reduced CYP3A5 activity might require lower sufentanil doses for comparable analgesia, highlighting the potential of genotype‐guided dosing [28]. However, for many CYP3A‐metabolized opioids, factors such as drug–drug interactions (DDIs) with potent inhibitors or inducers (e.g., antifungals, anticonvulsants, and macrolides), hepatic function, age, comorbidities, and concomitant treatments may be more clinically relevant than genotype alone [29–31]. Therefore, clinical recommendations regarding CYP3A4/5 genotype‐guided dosing should be approached with greater caution. While genetic testing can help stratify risk [32], routine dose adjustments based solely on CYP3A4/5 genotypes require more robust evidence and must be considered in the context of the specific drug and clinical scenario. Concurrent administration of therapies, particularly with CYP3A4/5 inhibitors, can further increase opioid bioavailability, exacerbating risks in individuals with low CYP3A4/5 expression [33].

#### 3.2.3. Future Directions

The growing understanding of CYP3A4/5 polymorphisms paves the way for personalized medicine. Future research should focus on developing reliable pharmacogenomic biomarkers to predict interindividual variability in response to fentanyl and sufentanil. Additionally, clinical trials are warranted to validate genotype‐guided dosing algorithms to ensure safer and more effective opioid therapy. Clinical recommendations for dose adjustments based specifically on CYP3A4/5 genotypes currently lack sufficient supporting evidence [34].

### 3.3. Mechanistic Overview of CYP‐Mediated Opioid Metabolism

**FIGURE 1:**
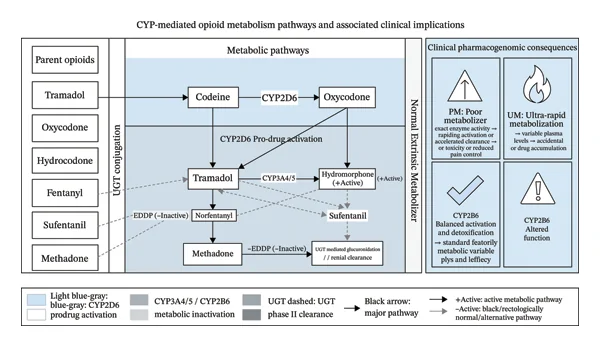
CYP‐mediated opioid metabolism pathways and associated clinical pharmacogenetic implications. Left panel lists commonly prescribed opioids. The central schematic illustrates major metabolic reactions catalyzed by Cytochrome P450 enzymes (CYP2D6, CYP3A4/5, and CYP2B6) and Phase II UGT enzymes. Green‐labeled routes denote CYP2D6‐dependent prodrug activation generating pharmacologically active metabolites, whereas gray pathways represent metabolic inactivation via N‐dealkylation. The right panel summarizes clinical outcomes linked to variable CYP genotypes, including poor metabolizers (PM) and ultrarapid metabolizers (UM): altered enzyme function results in insufficient analgesia or elevated risk of respiratory toxicity, necessitating personalized dose adjustment.

Elucidating the mechanisms by which Cytochrome P450 (CYP) enzymes metabolize opioids is crucial for predicting interindividual variability and optimizing therapeutic outcomes. CYP enzymes, particularly CYP3A4/5, CYP2D6, and CYP2B6, are pivotal in metabolizing various opioids [35]. Figure 1 illustrates the key metabolic pathways and clinical implications of CYP polymorphisms for opioid response (see Table 1).

**TABLE 1:** Common opioids, metabolic enzymes, and genetic typing guidance recommendations.

Opioids	Main metabolic enzymes	Related polymorphisms	Expected phenotypic effects	Clinical recommendations
Codeine	CYP2D6	^∗^PM, ^∗^UM	PM: analgesia ineffective; UM: toxicity risk (respiratory depression)	Avoid using in PM and UM
Tramadol	CYP2D6	^∗^PM, ^∗^UM	PM: analgesia ineffective; UM: potential toxicity risk	Avoid using in PM and UM
Oxycodone	CYP2D6, CYP3A4	^∗^PM, ^∗^UM	PM: analgesic efficacy may be reduced; UM: potential toxicity risk	Genotype is considered, but not the sole determining factor
Hydrocodone	CYP2D6	^∗^PM, ^∗^UM	PM: analgesic efficacy may be reduced; UM: potential toxicity risk	Genotype is considered, but not the sole determining factor.
Fentanyl	CYP3A4/5	Low‐expression group	Decreased drug clearance and increased risk of respiratory depression	Adjust the dosage with caution and monitor for drug interactions
Sulfentanyl	CYP3A4/5	Low‐expression group	Decreased drug clearance may necessitate a lower dose	The genotypic guidance dose should be considered, but additional evidence is required
Methadone	CYP2B6	^∗^6 alleles (PM)	Decreased drug clearance and elevated plasma concentration	Consider using a lower starting dose and closely monitoring the patient

*Note:* PM = slow metabolizer; UM = ultrarapid metabolizer.

#### 3.3.1. Genetic Polymorphisms and Interindividual Variability

Genetic polymorphisms in the genes encoding these CYP enzymes can significantly influence their activity. For instance:

• CYP2D6 PMs: Individuals with reduced CYP2D6 activity may experience diminished conversion of codeine to morphine, leading to inadequate analgesia [9].
• CYP3A4/5 Polymorphisms: Variations in CYP3A4 and CYP3A5 genes can affect the metabolism of fentanyl and sufentanil, altering their systemic exposure and efficacy [31].

#### 3.3.2. Role of DDIs

The activity of CYP enzymes can be modulated by concomitant medications.

• CYP3A4/5 Inhibitors: Drugs such as ketoconazole and clarithromycin can inhibit these enzymes, increasing systemic levels of fentanyl and sufentanil and consequently the risk of respiratory depression [36].
• CYP3A4/5 Inducers: Conversely, medications such as rifampin and phenytoin can induce CYP3A4/5 activity, accelerating opioid metabolism and potentially reducing efficacy [37].

#### 3.3.3. Clinical Implications and Future Directions

Elucidating the mechanistic roles of CYP enzymes is essential for developing personalized treatment strategies. Pharmacogenomic testing to identify relevant polymorphisms facilitates the tailoring of dosing regimens. Future research should focus on identifying novel biomarkers and developing predictive models to further enhance the precision of opioid therapy. It is important to distinguish between established associations and speculative future applications.

## 4. Genetic Polymorphisms and Clinical Outcomes

### 4.1. CYP2D6 Polymorphisms and Opioid Efficacy

Genetic polymorphisms in the CYP2D6 gene significantly influence the metabolism of several opioids, including codeine, tramadol, hydrocodone, and oxycodone, leading to different metabolic phenotypes (PM, IM, EM, and UM) that impact efficacy and safety.

#### 4.1.1. PMs

Individuals with genotypes such as CYP2D6 ^∗^4/^∗^4 (or other combinations of nonfunctional alleles) are classified as PMs. They exhibit reduced or absent CYP2D6 enzyme activity, leading to impaired metabolism of prodrugs such as codeine and tramadol. Since codeine requires CYP2D6 for conversion to its active metabolite, morphine, PMs may fail to achieve adequate pain relief [38]. Similarly, the bioactivation of tramadol into O‐desmethyltramadol is compromised in PMs, resulting in reduced efficacy [39]. Additionally, the accumulation of the parent drug may increase the risk of opioid‐related toxicity. Regulatory agencies such as the U.S. FDA have issued warnings regarding codeine use in PMs, especially children [11].

#### 4.1.2. UMs

Individuals with multiple functional copies of the CYP2D6 gene (e.g., gene duplications) are classified as UMs. They metabolize opioids at an accelerated rate, resulting in potentially excessive plasma concentrations of active metabolites. The strongest clinical evidence for CYP2D6 UM‐related severe toxicity involves codeine, where rapid conversion to morphine can increase the risk of adverse effects such as respiratory depression and sedation [12]. Notably, the strength of this association varies by drug and can be influenced by other factors. Rapid depletion of the parent drug might also occur, potentially leading to inadequate analgesia in some instances [40]. For example, UMs metabolizing tramadol may rapidly generate high levels of the active metabolite, increasing the risk of adverse effects while potentially undermining analgesic efficacy [25]. Extrapolation of these UM‐related toxicity concerns to other opioids, such as oxycodone or hydrocodone, warrants caution, as the role of CYP2D6 often involves generating active metabolites rather than being the sole pathway for the parent drug’s efficacy.

#### 4.1.3. Clinical Implications of CYP2D6 Polymorphisms

The variability in CYP2D6 activity has significant clinical implications:

• Therapeutic inefficacy: PMs may not achieve adequate pain relief with standard doses of prodrug opioids.
• Increased risk of toxicity: UMs may experience excessive opioid effects, increasing the risk of respiratory depression and overdose, particularly with codeine.
• Need for personalized dosing: Genetic testing for CYP2D6 polymorphisms can guide the selection of opioids and dosing strategies [28].

### 4.2. CYP3A4/5 Polymorphisms and Adverse Effects

The CYP3A4/5 gene family plays a critical role in the metabolism of opioids such as fentanyl, sufentanil, methadone, and oxycodone. Genetic polymorphisms can lead to variations in enzyme activity, influencing drug clearance, bioavailability, and the risk of adverse effects.

#### 4.2.1. High‐Expression Genotypes

Individuals with high‐expression genotypes (e.g., CYP3A4 ^∗^1/^∗^1 and CYP3A5 ^∗^1/^∗^1) exhibit increased enzyme activity, leading to potentially reduced systemic opioid exposure and decreased efficacy, requiring higher doses for adequate pain relief [25, 26]. However, this increased activity can also reduce the risk of toxicity by minimizing drug accumulation [41].

#### 4.2.2. Low‐Expression Genotypes

Conversely, individuals with low‐expression genotypes (e.g., CYP3A5 ^∗^3/^∗^3) exhibit reduced enzyme activity, leading to potentially increased drug bioavailability and a higher risk of adverse effects due to slow metabolism and drug accumulation [22, 23]. For example, a 2023 study in Pharmacogenomics found that individuals with low CYP3A5 expression had higher oxycodone concentrations, increasing sedation and respiratory depression risk [42].

#### 4.2.3. Clinical Implications of CYP3A4/5 Polymorphisms

The variability in CYP3A4/5 activity has clinical implications:

• Dose adjustment: Patients with high expression may require higher doses, while those with low expression may need lower doses. However, recommendations for dose adjustment based specifically on CYP3A4/5 genotype require more substantial evidence and must be considered in the context of the specific drug and clinical situation [34].
• Risk stratification: Genetic testing can help identify individuals at higher risk for adverse effects [32].
• Combination therapies: CYP3A4/5 inhibitors (e.g., ritonavir) can further increase opioid bioavailability, exacerbating risks in low‐expression individuals [33]. DDIs, hepatic function, age, comorbidities, and concomitant treatments are often more clinically relevant than genotype alone.

### 4.3. Racial Differences in CYP Enzyme Polymorphisms

The distribution of Cytochrome P450 (CYP) enzyme polymorphisms varies significantly across different racial and ethnic populations, contributing to interindividual differences in drug metabolism, efficacy, and safety. Understanding these variations is essential for advancing personalized medicine and optimizing treatment.

#### 4.3.1. Specific Examples of CYP Enzymes and Racial Differences

CYP2D6: The ^∗^4 allele is more prevalent in European populations (e.g., approximately 15%–20% in Caucasians), leading to a higher frequency of PM phenotypes. In contrast, the ^∗^10 allele (reduced function) is common in East Asian populations (e.g., up to 50% in some Chinese populations). The ^∗^5 allele (gene deletion) is also relatively common in some populations. This affects the metabolism of drugs such as antidepressants and opioids [43].

CYP3A4/5: The CYP3A5^∗^3 allele (associated with reduced expression) is more frequent in individuals of African descent (e.g., approximately 70%–80%) compared to Caucasians (e.g., approximately 10%–20%). These differences can impact drug clearance and pain management outcomes [41].

CYP2C9: Variations in this enzyme, which metabolizes warfarin, differ across racial groups [36].

#### 4.3.2. Causes of Racial Differences in CYP Polymorphisms

The causes are multifactorial, including genetic drift, evolutionary adaptations (e.g., to diet, pathogens), and environmental factors [44].

#### 4.3.3. Clinical Implications

These differences warrant the implementation of tailored dosing strategies to optimize outcomes and minimize adverse effects [38]. Integrating pharmacogenomic testing into practice holds promise but faces challenges such as cost, turnaround time, availability, and regulatory status. Current guidelines frequently continue to position pharmacogenomics as an option rather than a standard. Future research should focus on broadening the evidence base across diverse populations and developing inclusive clinical guidelines.

It is recommended to consult references providing quantitative allele frequency https://doi.org/10.1515/dmdi-2014-0023;https://doi.org/10.1371/journal.pone.0228000.

## 5. Clinical Implications and Future Directions

### 5.1. Evidence‐Based Recommendations and Future Directions

Integrating pharmacogenomic information into opioid prescribing has the potential to personalize therapy and improve patient outcomes. However, it is crucial to distinguish among guideline‐supported recommendations, pharmacokinetic associations, observational evidence, and speculative future applications.

Current evidence, particularly from the Clinical Pharmacogenetics Implementation Consortium (CPIC) and Dutch Pharmacogenetics Working Group (DPWG), supports the use of CYP2D6 genotype information in specific clinical scenarios.

1. CYP2D6 Genotype‐Guided Therapy:
• For prodrugs such as codeine and tramadol, avoiding use in CYP2D6 PMs is strongly recommended due to the risk of therapeutic failure [5, 6]. For CYP2D6 UMs, while increased risk of toxicity with codeine is a concern [10–12], recommendations for other CYP2D6‐metabolized opioids are less definitive and require cautious interpretation. The CPIC guideline on CYP2D6, OPRM1, and COMT genotypes and select opioid therapy provides comprehensive recommendations for various genotypes and opioid use [6]. Similarly, the DPWG guideline concerning the gene–drug interaction between CYP2D6 and opioids provides valuable insights [5].
2. CYP3A4/5 Genotype‐Guided Therapy:
• While polymorphisms in CYP3A4/5 can influence opioid metabolism, routine dose adjustments based solely on CYP3A4/5 genotype are generally not yet standard of care due to the significant influence of other factors such as DDIs, hepatic function, age, comorbidities, and concomitant treatments [31, 34]. Dose adjustments for CYP3A5 nonexpressors (characterized by low activity) may be considered, particularly for opioids such as fentanyl and sufentanil; however, this approach necessitates careful clinical judgment and monitoring [28].
3. CYP2B6 Genotype‐Guided Therapy:
• The CPIC guideline on CYP2B6 genotype and methadone therapy specifically addresses methadone management based on CYP2B6 genotypic status [15].

### 5.2. Implementation Barriers and Future Research Directions

Despite the growing body of evidence, several implementation barriers hinder the widespread adoption of pharmacogenomics in routine opioid prescribing [45]. These include the cost of genetic testing, turnaround time for results, limited availability of testing, lack of clinical decision support tools, and the need for clinician education.

Future research should focus on:

• Large‐Scale Multicenter Studies: Conducting large cohorts to validate the clinical utility of CYP genotype‐guided opioid therapy across diverse populations and clinical settings [45].
• Development of Novel Opioids: Designing opioids less dependent on polymorphic CYP enzymes could mitigate the impact of genetic variability.
• Integration of Multimodal Data: Combining genetic, pharmacokinetic, pharmacodynamic, and clinical data for a more comprehensive personalized approach.
• Addressing Implementation Challenges: Developing strategies to overcome cost, accessibility, and educational barriers to facilitate wider clinical adoption.

### 5.3. Evidence Grading and Nuance in Interpretation

It is imperative to clearly distinguish between:

• Guideline‐supported recommendations: Evidence‐based recommendations from organizations such as CPIC and DPWG.
• Pharmacokinetic associations: Studies demonstrating altered drug levels based on genotype, which may or may not translate to clinical outcomes.
• Observational evidence: Real‐world data suggesting correlations between genotype and clinical events, which can be prone to confounding factors.
• Speculative future applications: Emerging research or hypotheses that require further validation.

The strongest clinical evidence for CYP2D6 UM‐related severe toxicity concerns codeine. Extrapolation to other opioids should be presented more carefully [12]. For many CYP3A‐metabolized opioids, DDIs, hepatic function, age, comorbidities, and concomitant treatments may be more clinically relevant than genotype alone [31].

## 6. Conclusion

CYP enzyme polymorphisms are a significant determinant of individual differences in opioid response, substantially impacting both safety and efficacy in anesthesia and pain management. Personalized opioid therapy, informed by genetic data, holds promise for safer and more effective treatment. Current evidence supports selective pharmacogenomic testing in specific clinical scenarios, rather than broad routine implementation for all opioid prescribing.

### 6.1. Specifically

CYP2D6 genotyping is recommended for guiding the use of codeine and tramadol, with clear recommendations for PMs and considerations for UMs. For other CYP2D6‐metabolized opioids, genotype information can inform risk assessment.

For CYP3A4/5‐metabolized opioids, the influence of DDIs and other clinical factors often outweighs genotype alone, necessitating a cautious approach to genotype‐guided dosing. The integration of genetic information into clinical practice for opioid therapy holds great promise for advancing precision medicine in anesthesia and pain management. However, further research is needed to establish standardized protocols for genotype‐guided opioid therapy, to address implementation challenges, and to clearly distinguish between guideline‐supported recommendations, pharmacokinetic associations, observational evidence, and speculative future applications.

## Author Contributions

Rongrong Yan, Yingyao Quan, and Mengjiao Wan contributed substantially to the conception and design of the manuscript. Junyang Ma and Jing Cheng contributed to data acquisition, analysis, and interpretation. All authors participated in drafting the manuscript. Rongrong Yan revised it critically.

## Funding

This work was supported by the Hubei Natural Science Foundation—Maternal and Child Innovation and Development Fund (No. 2025AFD687, 2025AFD672) and Young Scientists Fund of the Maternal and Child Health Hospital of Hubei Province (No. 2023SFYY004).

## Disclosure

All authors read and approved the final version of the manuscript.

## Ethics Statement

Ethical approval was not required for this narrative review as it did not involve human or animal subjects.

## Consent

This is a narrative review article that synthesizes findings from previously published studies. All data and materials presented in this review have been appropriately cited. No original patient data or identifiable personal information requiring specific consent for publication are included.

## Conflicts of Interest

The authors declare no conflicts of interest.

## Data Availability

This review is based on previously published studies. All supporting data can be found in the reference list. No new datasets were generated.
